# Maternal physical activity levels in early pregnancy and the risk of spinal deformity among preschoolers at age 4: findings from the Shanghai birth cohort study

**DOI:** 10.3389/fped.2025.1639611

**Published:** 2025-08-26

**Authors:** Xin Li, Xuan Zhou, Juping Liang, Zefan Huang, Mengdie Jin, Xiuhua Shen, Xuanfang Wang, Binbin Fu, Peijie Chen, Qing Du

**Affiliations:** ^1^ School of Exercise and Health, Shanghai University of Sport, Shanghai, China; ^2^ Department of Rehabilitation, Xinhua Hospital, Shanghai Jiaotong University School of Medicine, Shanghai, China; ^3^ Children's Rehabilitation Innovation and Transformation Research Center of Yuanshen Rehabilitation Institute, Shanghai Jiaotong University School of Medicine, Shanghai, China; ^4^ Department of Clinical Nutrition, Xinhua Hospital, Shanghai Jiaotong University School of Medicine, Shanghai, China; ^5^ Ministry of Education and Shanghai Key Laboratory of Children's Environmental Health, Xinhua Hospital, Shanghai Jiaotong University School of Medicine, Shanghai, China

**Keywords:** cohort study, scoliosis, physical activity, pregnancy, preschool children

## Abstract

**Objective:**

To study the impact of maternal physical activity levels before and after birth on the risk of spinal deformity in preschool children.

**Methods:**

A cohort study of 760 preschoolers and their mothers tracked maternal physical activity levels during the prenatal period and the two years postnatally, as well as for two years after the child's birth, using standardized questionnaires at 6, 12, and 24 months. The risk of spinal deformity was assessed by the angle of trunk rotation (ATR) at the thoracic (T5), thoracic-lumbar (T12), and lumbar (L4) segments of the spine, with max values noted. An adjusted logistic regression model was used to explore the relationships between prenatal and postnatal physical activity levels and the risk of spinal deformity in preschoolers.

**Results:**

In 98 children (12.9%), ATRs were 3 or above, and 3 had ATRs of 5 at age 4. The duration of physical activity during early pregnancy (min/week) indicated a moderate risk of spinal deformity (with an ATR between 3 and 5) in children at age 4 (OR: 0.986, 95% CI: 0.976–1.001, *P* = 0.084). Exercising outdoors <1 h/day during 0–6 months reduced spinal deformity risk (3 ≤ ATRs < 5) compared to >1 h (OR = 0.525, 95% CI 0.301–0.917, *p* = 0.024). The risen risk of high ATR with long outdoor time was more significant when maternal blood calcium levels were low (OR=0.302, 95% CI 0.134–0.682; *p* = 0.004).

**Conclusion:**

Long outdoor times (>1 h/day) in infants under 6 months may be associated with changes in trunk rotation angle or postural stress. Exercise during early pregnancy may relate to good spine development in children. Further studies are needed on physical activity's role in scoliosis prevention.

## Introduction

Spinal health is a major global public problem associated with children, and scoliosis is the most common coronal plane spinal deformity in all childhood stages and can be screened early by measuring the angle of trunk rotation with the Adam test (1). Aggravated coronal curve deformity of the spine can lead to back pain (2), which is ranked 4th as the leading cause of years lived with disability in adolescents (3), resulting in a global economic stress burden that affects the quality of life throughout the lifespan and cannot be ignored.

Studies have reported that the etiology of spinal morphology starts early in life is multifactorial, with potential predisposing factors including bone development in perinatal, fetal and early infant stages (4–6). Recurrent low back pain and abnormal postural alignment in a 4-year-old child may be early manifestations of spinal disorders, which warrants attention (7).The formation of the spine begins during the embryonic period, and the spine continues to mature during the fetal period, so skeletal development during the fetal period has an important influence on the development of the spine after birth, affecting the structure and function of the spine after birth (8). The nutritional status of pregnant women, especially the level of blood calcium, has a direct impact on the formation of fetal bones (9, 10). In addition, studies have demonstrated that maternal lifestyle during pregnancy (physical activity, diet and body weight) is also crucial for fetal bone mineral density, influencing the increased risk of structural and functional abnormalities in bone during adulthood (11, 12). In the postnatal period, factors such as childhood nutrition (especially adequate calcium intake and vitamin D status) or physical activity contribute significantly to the bone health of the offspring. In infancy and childhood are characterized by a child's weakened muscle strength, poor stabilization and poor physiological curvature of the spine (13), which can be improved by habitual mechanical stimuli and physical activity. Studies have shown that bone quality and resistance to physical stress in childhood are largely dependent on habitual mechanical stimuli, while skeletal properties and postural development in childhood occur within a common, interrelated mechanical environment that may be modulated by specific patterns of anthropometry and body composition (14–16). Physical activity regulates the expression of muscle and bone cytokines to varying degrees to stimulate the development of muscles and bones (17). Preterm infants receiving 10 min of passive exercise three times per day experience 0.3% extra weight gain each day (18). For term infants between 2 and 4 months of age, fifteen minutes of passive exercise results in an increase of approximately 11.5 g/cm^2^ in bone mineral density (BMD) (19). A lack of proper exercise may lead to the underdevelopment of muscles and ligaments (20), which in turn affects the stability of the spine, prolonged exposure to poor posture may affect the normal growth of the spine, resulting in uneven spinal stress or asymmetrical growth (21). However, the relationship between lack of physical activity and spinal curvature in offspring is unclear, and what kind of physical activity is most beneficial for spinal morphology development in the preschool years remains unattended. To date no quantitative studies have examined this potential association.

The Shanghai birth cohort, which is an ongoing prospective study of health-influencing factors of child development (22). After signing informed consent forms, the recruited couples were followed up for at least 2 years after delivery. This study is a part of the Shanghai birth cohort, formulating a follow-up plan for children aged 4 and inviting these families to participate. It aims to examine the potential cumulative effect of the physical activity levels and blood calcium levels during the maternal prenatal period and child's first 2 years on the risk of spinal deformity among preschoolers at age 4.

## Material and methods

### Study design and participants

This study is a prospective cohort study, which utilized data from the Shanghai birth cohort, which enrolled couples planning pregnancies or in early stages of pregnancy from six major Shanghai hospitals. Participants were required to be long-term residents, and the cohort was established in 2014 with ongoing follow-ups of the children. Due to the complexity and radiation of x-rays, spinal deformity was defined as the risk of altered spinal morphology in the coronal plane detected by scoliosis screening, as recommended by the US Preventive Services Task Force (USPSTF) (23). The Adam forward bending test was performed for scoliosis screening for the purposes of reducing economic costs and minimizing radiation exposure (1). Out of 1,487 families, 768 with complete data underwent the Adam's forward bend test and were included. The study was approved by Xinhua Hospital's Medical Ethics Committee (XHEC-C-2013-001). Excluding children with a family history of scoliosis (*n* = 4), inherited metabolic disorders (*n* = 3), and central nervous system disorders (*n* = 1), the final sample comprised 760 families.

### Data collection

#### Follow-up flow

The family would be reminded to order an offline follow-up visit in Follow-up Center of the Shanghai birth cohort when children reached 6, 12, 24, or 48 months. Mothers and children would attend the follow-up visit. We encourage other caregivers to participate as well if the mother was not the main caregiver. For children, one mineral density was measured at each visit. Body parameter measurements and scoliosis screening were conducted at age 4. For caregivers, they would be asked to fill out questionnaires about recent child care at each visit.

### Scoliosis screening

The Adam forward bending test was performed for scoliosis screening, and the angle of trunk rotation (ATR) was measured by the Bunnell scoliometer (24). Researchers must be qualified as rehabilitation physicians and complete training in Bunnell scoliometer usage. The children were asked to stand relaxed, keep their palms together, and bend their trunks forward. One researcher demonstrated the proper posture beforehand and helped the children to keep their feet together and their legs straight. Then, the researcher centered the Bunnell scoliometer using the spinous process, measured the tilt at the thoracic (T5), thoracolumbar (T12), and lumbar (L4) segments of the spine, and recorded the maximum value of the three segments as the ATR. An ATR value of 5 or above was defined as a positive screening result. Another researcher monitored the child's posture throughout the test and made a repeated test call if the child did not maintain the correct posture. Most of the children completed the screening test, which lasted approximately 30 s. Children who failed to cooperate during tests were excluded (1, 25, 26).

### Children's screen time and physical activity

Information on children's activities, including screen time, indoor and outdoor activity, was also collected through three specific questions at 6 months, 12 months, and 24 months of age, respectively. Screen time was described as “no screen time”, “0–60 min per day”, or “over 60 min per day”. Indoor activity required intimate contacts between children and caregivers in indoor environments. It was grouped by “every day” (7 days in a week), “most days” (4–6 days in a week) or “seldom” (3 or less than 3 in a week). Time that children spent outdoors with the supervision of their caregivers per day was collected and divided into two groups based on a duration of 60 min per day.

### Children’s body parameter measurements

Includes height, weight, body fat ratio, muscle mass, sitting height and bone mineral density. Height and sitting height were measured through a wall-mounted meter. When measuring sitting height, the children sat on a 25 cm high stool, keeping their hip, scapulae, and head against the wall. Weight and body fat were measured by bioelectrical impedance analysis through InBody720 (InBodyUSA, Cerritos, CA). The bone mineral density of the left tibia was measured by quantitative ultrasound (Omnisense 7000P, Sunlight Medical Inc., Israel). Height and sitting height measurements would be repeated two times to get arithmetic means. Other body parameters were measured once.

### Maternal physical activity and serum trace element levels during pregnancy

Mothers self-reported their daily moderate-to-vigorous-intensity activity time and activity frequency during pregnancy through a culturally adapted version of the International Physical Activity Questionnaire (IPAQ). In addition, maternal serum trace element levels during pregnancy were obtained from a blood sample taken during the first prenatal visit during the first trimester.

### Statistical power

The statistical power check was based on the results of Cai et al. on scoliosis and activity duration (27). They categorized 2457 children age 6–12 into 4 × 2 contingency table based on daily physical activity duration and ATR value. Their results showed that children with less than 1 h of activity per day (OR = 7.29, 1.99–53.37) and with 1–2 h activity per day (OR = 7.09, 1.09- 52.17) exhibited a higher risk of scoliosis compared to those with more than 3 h/day. Based on the data distribution in Cai's contingency table, we calculated an effect size W of 0.59. The statistical power test was based on the existing sample size and the calculated effect size W, total sample size = 760, W = 0.59, set a = 0.05, the power of the Chi-square test was calculated as 0.99 > 0.95.

### Covariates

The following variables were collected during pregnancy from medical records: maternal age, mode of delivery, birth weight, gestational age, and serum Ca level during pregnancy.

### Statistical analysis

We compared parameter characteristics between the normal and high ATR groups. Continuous data are mean ± SD, and categorical variables are numbers (percentages). T-tests, chi-square, and Kruskal–Wallis tests were applied. The missing data proportion was within 5%, which can be considered as missing completely at random (MCAR), and listwise deletion was applied for handling. Logistic regression was used to estimate the interaction between ATR and physical activity during pregnancy and postpartum, with crude and adjusted estimates for covariates like early pregnancy blood calcium, bone mineral density at age 4, and sex. The odds ratio (OR) and 95% confidence interval (CI) for high ATR were reported. IBM SPSS Statistics 25 (IBM, Inc., USA) was used, with *P* < 0.05 indicating significance.

## Results

A total of 760 families composed of mothers and children with complete data were included in the study after exclusions. Of these, 98 children (12.89%) with ATRs of 3 or higher were categorized as high risk for scoliosis, while 662 children (87.11%) with ATRs of 0–2 were considered low risk. Within the high ATR group, 3 children (0.39%) had ATRs of 5 or above, 10 children (1.32%) had ATRs of 4, and 84 children (11.05%) had ATRs of 3. Table 1 presents the demographic and perinatal history characteristics, and there was no significant difference between the high-ATR group and the normal-ATR group. Results of the normality test for demographic characteristics are shown in Supplementary Table S1.

**Table 1:** Distribution of ATR value.

ATR value	Paticipants (No.)	Percentage
0	56	7.4%
1	314	41.3%
2	292	38.4%
3	84	11.1%
4	11	1.4%
5 or above	3	0.4%

ATR, angle of rotation.

Parents usually pay a great deal of attention to children's physical activity after birth, with more than 90% of children in all visits reporting performing indoor activities every day. We therefore combined “most days” and “seldom” in the analysis (Table 2). In contrast, during early pregnancy, only 12.1% of mothers performed regular physical activity. According to the univariate analysis, children under 6 months of age with 0–60 min of outdoor time per day had lower ATR values at age 4 than did those with more than 60 min of daily outdoor time (OR = 0.581, 95% CI 0.382–0.958, *P* = 0.033). A similar association was found in multivariate analysis when blood calcium levels in early pregnancy, bone mineral density at age 4, and sex were adjusted (OR = 0.491, 95% CI 0.283–0.854; *P* = 0.012). Moreover, physical activity during early pregnancy tended to decrease the ATR in offspring, although the difference was not significant (OR = 0.989, 95% CI 0.976–1.001; *P* = 0.084) (Table 3).

**Table 2:** Demographic characteristics of children and their mothers.

Characteristics	Total, *n* = 760	ATR 0–2, *n* = 662	ATR ≥ 3, *n* = 98	*P*
Child characteristics				
Gender				0.654
Boy	395	342 (86.6)	53 (13.4)	
Girl	365	320 (87.7)	45 (12.3)	
Birth weight (g)	3,375 (527.5)	3,380 (535)	3,350 (520)	0.776
Weight at age 4 (kg)	17.2 (3.1)	17.2 (3.1)	16.9 (2.6)	0.755
Body fat at age 4 (%)	13.3 (7.8)	13.4 (7.7)	12.6 (7.2)	0.277
BMI at age 4 (kg/m^2^)	14.8 (1.8)	14.8 (1.7)	14.7 (1.8)	0.265
Height at age 4 (cm)	108 (6)	108 (6)	109 (5)	0.231
Sitting height at age 4 (cm)	60.5 (4.5)	60.5 (4.5)	60.5 (3.5)	0.469
Bone density Z score at 6 m	−2.60 (1.95)	−2.60 (2.05)	−2.70 (1.95)	0.438
Bone density Z score at 12 m	−1.20 (1.3)	−1.20 (1.3)	−1.20 (1.15)	0.946
Bone density Z score at 48 m	−0.49 (1.25)	−0.49 (1.24)	−0.53 (1.03)	0.852
Maternal characteristics				
Maternal age at pregnancy (years)	30 (5)	29 (4)	30 (5)	0.177
Gestational Weeks (weeks)	39 (2)	39 (2)	39 (2)	0.766
Serum Ca during pregnancy (mg/dl)	85.8 (16.2)	85.9 (16.3)	85.5 (15)	0.685
Smoking history				0.092
Yes	17	12 (70.6%)	5 (29.4%)	
No	742	649 (87.5%)	93 (12.5%)	
Way of delivery				0.855
Spontaneous	422	367	55	
Cesarean	318	278	40	
Regular phycical activity during pregnancy				0.536
Yes	662	580 (87.6)	82 (12.4)	
No	98	88 (89.8)	10 (10.2)	

Data presented were mean ± sd for continue variables obey normal distribution, median (interquartile range) for continue variables disobey normal distribution, number (percentage) for categorical variables.

**Table 3:** Association between physical activity and ATR value at age 4.

Physical activity	*N* (ATR ≥ 3)	Crude OR (95% CI)	*P* ^b^	Adjusted OR (95% CI)^b^	*P* ^b^
Physical activity time during early pregnancy (min/week)	760 (98)	0.997 (0.993–1.001)	0.179	0.989 (0.976–1.001)	0.084
0–6 m					
Indoor activity					
Every day	705 (89)	Referent		Referent	
Not every day	50 (8)	1.177 (0.719–1.927)	0.516	1.353 (0.805–2.274)	0.254
Daily outdoor time					
Over 60 min/day	450 (61)	Referent	–	Referent	–
0–60 min/day	305 (37)	0.581 (0.352–0.958)	0.033^c^	0.491 (0.283–0.854)	0.012^c^
Screen time					
No screen time	693 (90)	Referent	–	Referent	–
0–60 min/day	53 (7)	1.020 (0.447–2.328)	0.963	1.257 (0.455–3.470)	0.659
Over 60 min/day^a^	3 (0)	–	–	–	–
6 m−12 m					
Indoor activity					
Every day	681 (88)	Referent		Referent	
Not every day	63 (9)	0.996 (0.551–1.801)	0.991	0.792 (0.364–1.720)	0.556
Daily outdoor time					
Over 60 min/day	557 (71)	Referent		Referent	–
0–60 min/day	203 (27)	1.133 (0.694–1.852)	0.617	1.207 (0.661–2.205)	0.540
Screen time					
Near 0	580 (77)	Referent	–	Referent	–
0–60 min/day	159 (20)	0.940 (0.555–1.591)	0.818	1.133 (0.593–2.166)	0.706
Over 60 min/day^a^	9 (1)	–	–	–	–
12 m–24 m					
Indoor activity					
Every day	693 (89)			Referent	
Not every day	67 (9)	1.053 (0.504–2.200)	0.891	1.176 (0.496–2.785)	0.713
Daily outdoor time					
Over 60 min/day	568 (75)	Referent		Referent	–
0–60 min/day	192 (23)	1.182 (0.679–1.841)	0.662	1.229 (0.650–2.626)	0.526
Screen time					
Near 0	120 (12)	Referent	–	Referent	–
0–60 min/day	485 (64)	0.861 (0.489–1.517)	0.605	0.864 (0.418–1.786)	0.693
Over 60 min/day	147 (16)	0.692 (0.336–1.424)	0.317	0.726 (0.291–1.808)	0.491

^a^ Do not enter the model because of the small quantity.

^b^ Adjust for blood calcium of mons in early pregnancy, bone mineral density at age 4 and children’s gender.

^c^ The result is significant at level of 0.05.

**Figure 1:**
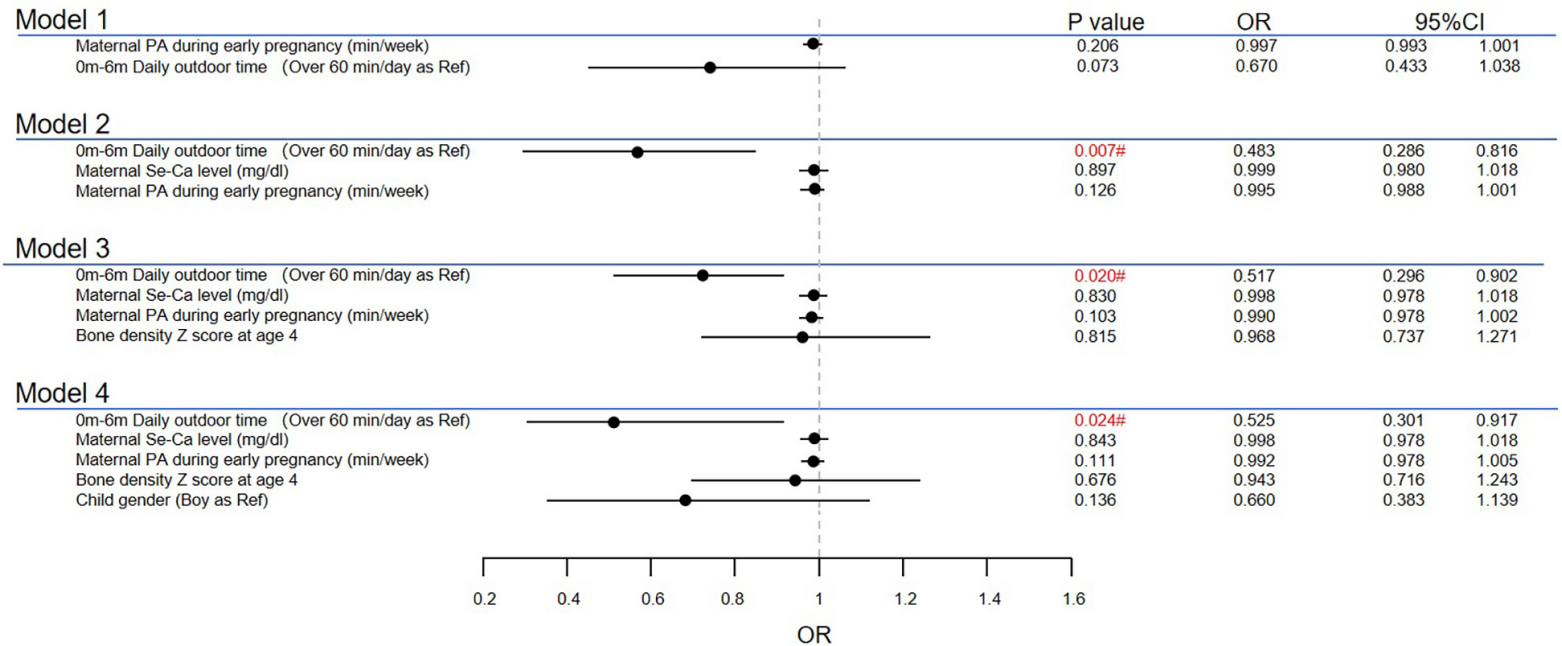
Multiple analyses for high ATR at age 4. #The result is significant at level of 0.05. PA, physical activity.

According to stepwise multivariate analysis (Figure 1), the model included physical activity time during pregnancy and daily outdoor time from 0 to 6 months after birth. Lower ATR values were found in children with short outdoor time in the age of 0–6 months when maternal serum Ca levels were adjusted (OR = 0.483, 95% CI 0.286–0.816; *P* = 0.007). This significant difference still existed after adding bone density and child sex to the regression model (OR = 0.525, 95% CI 0.301–0.917; *P* = 0.024). The relationship between maternal physical activity duration and the ATR was not statistically significant. No significant collinearity was found among the variables (Supplementary Table S2). Exploration of interaction effects was not conducted, as only the main effect of one variable was statistically significant.

**Figure 2:**
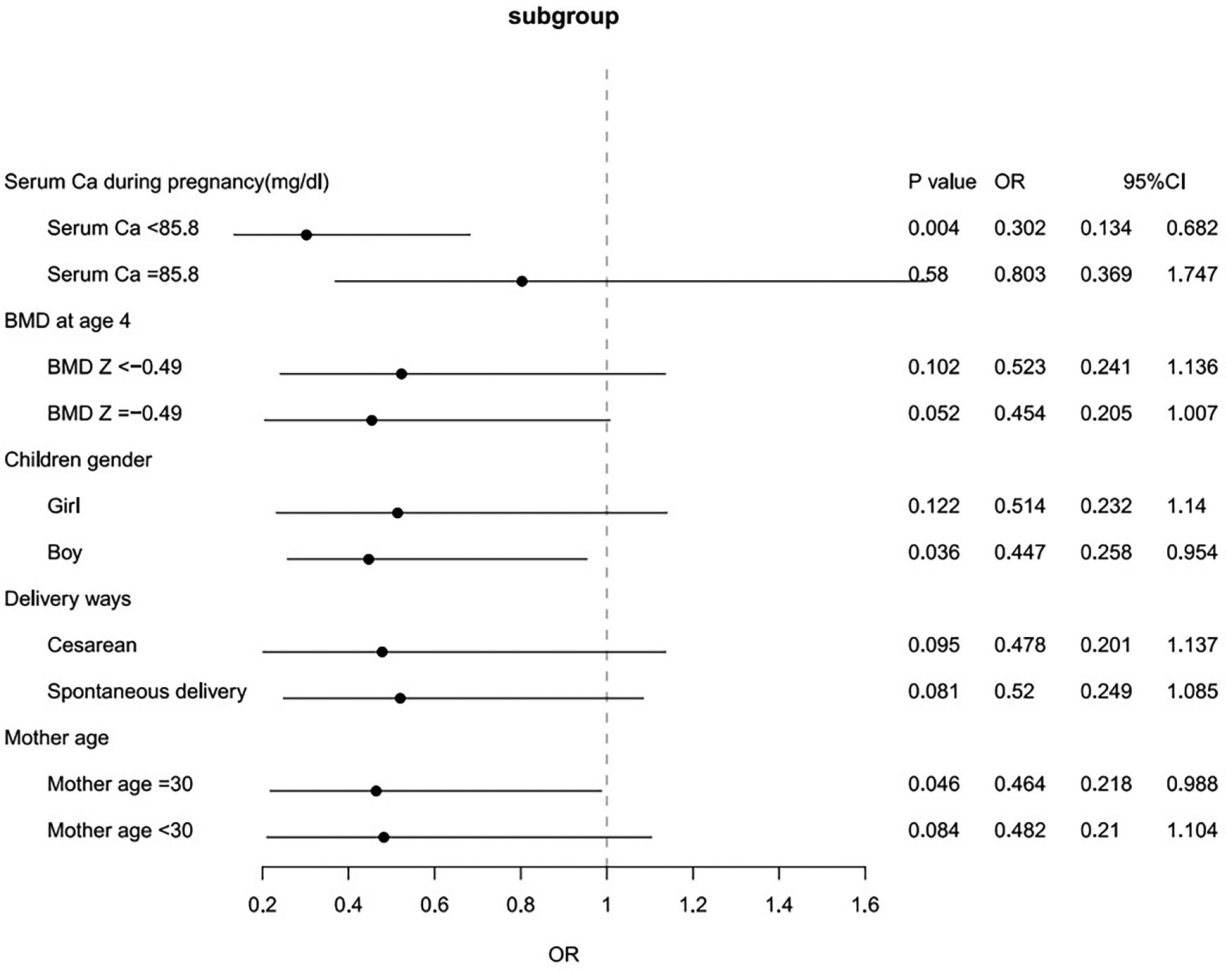
Subgroup analyses for the associations between short outdoor time at 0–6 months of age and ATRs.

We further conducted subgroup analyses based on maternal age (<30 vs. ≥30 years old), type of delivery (cesarean vs. normal delivery), maternal serum Ca level (≥85.8 mg/dl vs. <85.8 mg/dl), bone density Z score (≥−0.49 vs. <−0.49) and child sex. In offspring from mothers with low serum calcium levels, an outdoor time of less than 60 min per day significantly decreased the risk of high ATRs (OR = 0.302, 95% CI 0.134–0.682; *P* = 0.004) (Figure 2).

## Discussion

This study demonstrated that an outdoor activity time of more than 60 min per day in infants aged 0–6 months may have a relationship with high ATR at 4 years of age, especially when mothers had a low blood level of calcium during early pregnancy. Moreover, maternal physical activity tended to decrease the ATR in offspring, according to univariate analysis. No relationship was found between physical activity and the ATR or between sedentary behavior, represented by screen time, and the ATR in children aged 6–24 months.

A correlation between scoliosis and bone metabolism has been reported previously. Our previous study showed that children with scoliosis have a risk of low bone density that is approximately 7 times greater than that of healthy children (28). Bone development is a process of accumulation, with a peak in puberty (29). Although the effect of differences in bone density at age 4 was not significant in this study, it might still contribute to the incidence of scoliosis in adolescents, which needs further research. Attention should be given to bone strength monitoring and bone quality improvement in children. Our findings showed that the average Z score of BMD in the enrolled children was −1.12 at 12 months of age and −0.36 at 24 months of age. This significant increase indicated the effectiveness of nutritional supplements in improving bone development, which was consistent with previous research. Moreover, unlike the higher incidence of scoliosis in girls, no difference was found in the rate of high ATRs between boys and girls in this study. In early childhood, studies have shown similar bone density and bone metabolism conditions in boys and girls (30, 31). This result is consistent with the bone development course in children and indicates that sex is not the main factor affecting ATRs at an early age.

### Outdoor activities in early age, good or not?

Outdoor activities are generally closely related to physical exercise. Studies have established that exercise or physical activities can promote bone formation and subsequently prevent scoliosis even in infants from the perspectives of biomechanics and metabolism. Mechanical loads caused by exercise stimulate osteoblasts and activate their differentiation and mineralization (32, 33). In this process, the serum levels of inflammatory cytokines such as interleukin-6 (IL-6) and tumor necrosis factor-α (TNF-α) decrease, which inhibits bone resorption (34). Previous meta-analyses have shown moderate benefits for bone growth and weight gain in infants (35), and a cohort study revealed a negative correlation between motor ability at age 18 months and the risk of scoliosis at age 15 years (36). Our results showed that long periods of outdoor activity before 6 months of age might relate to high ATRs, which is not consistent with the findings of previous studies. Our study initiated follow-up from birth to 4 years old, covering earlier age groups, which may account for the divergent findings. We put forward the following two hypotheses to discuss the divergence.

### Damage from exercise

For infants aged 0–6 months, the risk of damage from exercise should not be neglected. There is a temporary decrease in the bone mineral density of infants from 0 to 6 months after birth, which is mainly due to adaptation to the extralateral environment and rapid bone marrow formation. During this period, the osteocyte network monitors the bone deformation caused by mechanical stimulation and adjusts the bone structure. Excessive mechanical stimulation, which is related to improper posture, may cause unbalanced bone development, including plastic deformation of the spine and pseudo subluxation of small joints (37, 38).

### Poor postures

According to Australian and Canadian movement guidelines, floor-based games are recommended for infants under 6 months due to the insufficient load-bearing capacity of their spine (39, 40). These games require a clean, comfortable, and safe environment, which may not be met under outdoor conditions. Based on this, we assume that infants under 6 months of age may usually stay in their parents’ arms or strollers. From this perspective, the time spent outdoors for infants under 6 months should not be recognized as physical activity time but rather as time spent staying in improper posture. Long-term poor posture may be a detrimental factor for spinal development. Compared to lying prone, being in the arm, car seat, or babysitter decreased the level of cervical paraspinal and erector spinae muscle activity, according to the results of surface electromyography (41). A long-term low level of paraspinal muscle activity results in a lack of mechanical loads toward the spine, which is closely related to abnormal ossification and tissue loss in joint regions (42).

### Potential dual-hit effects: low calcium during pregnancy and external shocks after birth

During embryonic development, essential minerals such as calcium and phosphate are maintained at high levels in the uterus and enter the fetus's circulation through the placenta to complete bone formation (43). Insufficient and low-quality evidence indicates that maternal calcium levels affect children's bone formation and mineralization, possibly because maternal factors are masked by postpartum factors (44). This study showed that children whose mothers had low blood calcium levels were susceptible to negative effects from prolonged outdoor activity at 0–6 months when stratified according to the mother's blood calcium level. Subclinical bone loss might occur in offspring from mothers with low serum calcium levels, leading to vulnerability to external shocks.

### Encouragement for activities during pregnancy

Previous studies have shown that exercise during pregnancy can promote placental function by enhancing placental blood flow, strengthening maternal-fetal connections, and promoting fetal development, which is similar to our result (45, 46). However, only 12.6% of pregnant women performed regular physical activity, which was not enough to reach a significant result. According to The American College of Obstetricians and Gynecologists, 150 min of moderate-intensity physical activities such as jogging and cycling per week are strongly recommended during pregnancy for the health of mothers and their offspring, particularly under conditions of low overall activity levels (47).

### Limitations

There were several limitations in this study. First, the children in the study were evaluated at 4 years of age. The screening results at this time might not fully represent the occurrence of scoliosis at school age or during puberty. Follow-up visits will continue at 7 and 10 years of age in the SBC study. Second, the source of the data was mainly questionnaires based on subjective recall with semiquantitative options, which might cause bias in the results. Thirdly, the findings should be interpreted cautiously due to residual confounders like genetic/epigenetic factors, environmental differences (e.g., urban-rural settings, pollution, sunlight), and parental care behaviors (e.g., carrying methods, stroller use), requiring more comprehensive adjustments in future studies. Fourth, extensive sample exclusion occurred during the enrollment process, primarily due to missed follow-ups. The excluded subset of participants may exhibit characteristics such as low adherence, limited parental engagement, or lower prioritization of child health, which reduces the generalizability to the broader population. Additionally, their generalizability to non-Chinese or low-income populations may be limited by cultural, lifestyle, nutritional, healthcare, environmental, and socioeconomic differences. Thus, future studies should use quantitative methods such as accelerometers to measure activity levels, and multicohort studies with diverse populations are needed to validate the results and assess their broader applicability.

In conclusion, this prospective cohort study revealed that children staying outdoors for children unable to maintain their spine upright may have an adverse effect on spinal development. Children at this age are suggested to mainly engage in floor activities and stay prone. Physical activity in early pregnancy may be beneficial to the development of the spine in offspring, while low blood calcium levels during pregnancy might have a relationship with increased susceptibility to damage.

## Data Availability

The data that support the findings of this study are available from the corresponding author upon reasonable request.
