# Supplementary material for: Maternal physical activity levels in early pregnancy and the risk of spinal deformity among preschoolers at age 4: findings from the Shanghai birth cohort study
Source: Front Pediatr. 2025 Aug 26;13:1639611. doi: 10.3389/fped.2025.1639611 (PMC12428481; doi:10.3389/fped.2025.1639611)
Supplement: Supplementary file 1 [file Supplementaryfile1.docx]

**Table S1** **Normality test for demographic characteristics.**

| Continuous characteristics | D value (K-S test) | P |
| --- | --- | --- |
| Child characteristics |  |  |
| Birth weight (g) | 0.034 | 0.043 |
| Weight at age 4 (kg) | 0.086 | <0.001 |
| Body fat at age 4 (%) | 0.243 | <0.001 |
| BMI at age 4 (kg/m^2^) | 0.099 | <0.001 |
| Height at age 4 (cm) | 0.070 | <0.001 |
| Sitting height at age 4 (cm) | 0.107 | <0.001 |
| Bone density Z score at 6m | 0.136 | <0.001 |
| Bone density Z score at 12m | 0.089 | <0.001 |
| Bone density Z score at 48m | 0.058 | <0.001 |
| Maternal characteristics |  |  |
| Maternal age at pregnancy (years) | 0.106 | <0.001 |
| Gestational Weeks (weeks) | 0.212 | <0.001 |
| Serum Ca during pregnancy (mg/dl) | 0.059 | <0.001 |

**Table S2 Multiple analyses of risk factors for high ATR at age 4.**

|  | High ATR at age 4 | OR (95% CI) | P | VIF |
| --- | --- | --- | --- | --- |
|  | Maternal PA during early pregnancy (min/week) | 0.997(0.993 to 1.001) | 0.206 | 1.001 |
| Model 1 | Daily outdoor time (child age 0 m-6 m) |  |  | 1.001 |
|  | Over 60 min/day | Ref | - |  |
|  | 0-60 min/day | 0.670 (0.433 to 1.038) | 0.073 |  |
|  | Maternal PA during early pregnancy | 0.995(0.988 to 1.001) | 0.126 | 1.007 |
| Model 2 | Daily outdoor time (child age 0 m-6 m) |  |  |  |
|  | Over 60 min/day | Ref | - |  |
|  | 0-60 min/day | 0.483 (0.286 to 0.816) | 0.007^*^ | 1.001 |
|  | Maternal Se-Ca level (mg/dl) | 0.999 (0.980 to 1.018) | 0.897 | 1.007 |
|  | Maternal PA during early pregnancy (min/week) | 0.990(0.978 to 1.002) | 0.103 | 1.011 |
| Model 3 | Daily outdoor time (child age 0 m-6 m) |  |  | 1.005 |
|  | Over 60 min/day | Ref | - |  |
|  | 0-60 min/day | 0.517 (0.296 to 0.902) | 0.020^*^ |  |
|  | Maternal Se-Ca level (mg/dl) | 0.998 (0.978 to 1.018) | 0.830 | 1.013 |
|  | Bone density Z score at age 4 | 0.968 (0.737 to 1.271) | 0.815 | 1.010 |
|  | Maternal PA during early pregnancy (min/week) | 0.992(0.978 to 1.005) | 0.111 | 1.016 |
| Model 4 | Daily outdoor time (child age 0 m-6 m) |  |  | 1.013 |
|  | Over 60 min/day | Ref | - |  |
|  | 0-60 min/day | 0.525 (0.301 to 0.917) | 0.024^*^ |  |
|  | Maternal Se-Ca level (mg/dl) | 0.998 (0.978 to 1.018) | 0.843 | 1.013 |
|  | Bone density Z score at age 4 | 0.943 (0.716 to 1.242) | 0.676 | 1.021 |
|  | Child gender |  |  | 1.024 |
|  | Boys | Ref | - |  |
|  | Girls | 0.660 (0.383 to 1.139) | 0.136 |  |
